# Teledermatology for Older Adults With a Focus on Nursing Home Residents: A Scoping Review of Clinical and System-Level Benefits

**DOI:** 10.7759/cureus.102891

**Published:** 2026-02-03

**Authors:** Julia L Armstrong, Salma Bennis, Jessica N Smock, Marc M Kesselman

**Affiliations:** 1 Dermatology, Nova Southeastern University Dr. Kiran C. Patel College of Osteopathic Medicine, Davie, USA; 2 Rheumatology, Nova Southeastern University Dr. Kiran C. Patel College of Osteopathic Medicine, Davie, USA

**Keywords:** assisted living, diagnosis, elderly, nursing home, quality of life, remote dermatologic care, store-and-forward dermatology, teledermatology, telehealth, virtual dermatology

## Abstract

Teledermatology (TD), which involves providing dermatology services, including diagnosis and management, remotely, has grown as a result of the COVID-19 pandemic, becoming a critical tool for delivering dermatologic care, especially to aging populations. Specifically, for nursing home residents who often face mobility and cognitive limitations, multimorbidity, and an increased risk of complications, TD may allow for earlier diagnoses, improved access to care and quality of life, and timely management. A scoping review of studies published between 2015 and 2025 was conducted to evaluate clinical and system-level outcomes. A comprehensive search was conducted by three independent researchers using multiple databases, including Ovid MEDLINE, EMBASE, and Web of Science. To analyze the most common dermatologic diagnoses in nursing homes, the inclusion criteria included geriatric patients (>60 years old), nursing home patients, and studies published in English between 2015 and 2025. For analyzing the overall benefits of using TD, the inclusion criteria were identical except that dermatology patients of any age were eligible. Exclusion criteria for analyzing the most common dermatologic diagnoses in nursing homes and the benefits of using TD included articles that were older than 15 years and case reports. Overall, this review will provide a comprehensive analysis of the benefits of using TD as a diagnostic and management tool for dermatologic conditions in the elderly nursing home setting.

## Introduction and background

Telehealth, or telemedicine, which is the use of online technology to deliver remote healthcare, has been increasing in recent years. As a result, specific subsets of telehealth, including teledermatology (TD), the use of telecommunication technology to remotely deliver dermatologic diagnosis and management, have been shown to improve patient outcomes and quality of life (QoL), especially for aging patients residing within nursing homes. TD allows for the remote consultation and diagnosis of patients with skin conditions, which can improve access to care by reducing the need for transport, costs tied to transport, infection exposure, and in-person wait times [1].

Extensions of TD have emerged to enhance clinical diagnosis, including teledermoscopy (dermoscopic images transmitted electronically), teledermatopathology (digitized histopathology images for remote skin biopsy readings), and telecytology (remote interpretation of cellular samples through digitized photos) [2-4]. Telemedicine has a long history in dermatology. The first reported instance of TD occurred in 1993 in Norway, where telemedicine was rapidly growing among highly visual medical specialties. As for its use in the US, TD began in 1995 in medically underserved populations of rural Oregon. The high frequency of skin diseases, coupled with the relatively small number of specialists in these rural areas, made the demand for dermatologists particularly high, furthering the rise of TD. The development of TD in the US was also advanced by the US Army Medical Department, which utilized TD for several decades to deliver specialized care to US troops in remote settings [5].

The accuracy of TD was first evaluated in Minnesota in 1997 in a study comparing diagnoses made by in-person dermatologists and teledermatologists among nursing home patients, demonstrating an 88% diagnostic concordance [6]. Since then, TD has expanded significantly and improved through the implementation of various confidentiality and security guidelines [7].

TD can be performed by licensed dermatologists or general practitioners to triage or follow up with patients, consult with other colleagues, or educate junior healthcare professionals. TD modalities can be broadly categorized into three types: (1) store-and-forward TD (SAF-TD): utilizes asynchronous still digital image technology for communication [8]; (2) real-time TD (RT-TD): live-video consultation; and (3) hybrid: a combination of both, characterized by image submission followed by real-time video interaction [9].

A summary of these modalities is shown in Table 1.

**Table 1:** TD modalities RT-TD, real-time teledermatology; SAF-TD, store-and-forward teledermatology; TD, teledermatology

Modality	Definition
SAF-TD/asynchronous	SAF-TD is an asynchronous model that stores patient images and history and transmits them to a dermatologist for evaluation later. High-quality photographs are sent to a secure platform, allowing for enhanced triaging and the ability for dermatologists to review cases without a real-time interaction [7].
RT-TD	RT-TD includes a live video interaction between patient and provider [9].
Hybrid	Hybrid connects elements of both SAF-TD and real-time by collecting patient images and data, followed by setting up a live video interaction [10].

Despite limited diagnostic accuracy for pigmented skin lesions, SAF-TD is a cost-effective and easy-to-use remote consultation method with notable value in dermatological practice, especially for nursing home patients who cannot easily attend a dermatology clinic [8]. RT-TD using live video technology is another one of the most frequently utilized types.

While TD has evolved over recent years, several initial complications required troubleshooting. One of the primary concerns with TD was the Health Insurance Portability and Accountability Act (HIPAA) of 1996 compliance. The use of remote mobile technologies posed a security risk to patient information and confidentiality, underscoring the importance of using secure platforms with data encryption, login controls, and other features required by the American Telemedicine Association Practice Guidelines [11]. An additional barrier was that TD initially left gaps in patients’ healthcare, as consultation records were rarely sent to patients’ primary care providers [11].

Live dermatology consultations in nursing homes have historically been limited due to the overwhelming demand for long-term care and shortages of dermatologists available for in-person visits. In addition, nursing home residents often have functional or cognitive impairment, along with transportation and mobility limitations, making in-person dermatology clinic visits difficult.

TD has the potential to reduce unnecessary in-person visits by identifying cases that may be managed remotely. One common reason for dermatology consultation in nursing homes is the presence of skin tumors (benign and malignant) [12]. Patients with skin tumors experience an added value from additional live consultations so that diagnostic and therapeutic procedures (such as lesion palpation, dermoscopy, punch biopsy, cryotherapy, and shave excisions) can be performed. With TD, if skin cancers are detected, planning for a biopsy, either in the nursing home or at the dermatologist’s office, needs to be arranged. Many other dermatologic conditions seen in nursing homes, such as eczema, (pressure) ulcers, skin infection, psoriasis, pruritus, xerosis, and bullous skin diseases, do not always warrant a live consultation or in-person visit. As such, TD and live consultations can serve as a complement to one another, enabling more efficient care in settings where in-person evaluations can be difficult.

Although the development of telehealth can be traced back 20 years, its value (and quick expansion) was accelerated during the COVID-19 pandemic. Considering savings in personal protective equipment and in-office materials, cost concerns became less of a barrier [13]. In addition, patient and physician outlooks on virtual visits evolved, with both groups demonstrating wider acceptance [13]. During the COVID-19 public health emergency, many states implemented temporary regulatory waivers, including modifications to licensure requirements, to facilitate telehealth delivery [14]. These changes enabled timely diagnosis and management of new and chronic conditions and were particularly impactful for patients who face significant barriers to in-person specialty care, such as nursing home patients [14].

Telehealth in dermatology shows promise not only in improving health outcomes but could also play a role in improving patients’ QoL, especially in the geriatric patient population. A 2013 study involving dermatology patients over the age of 60 found that 62.3% of these patients had a geriatric depression score (GDS) over 10 compared to the general population, in which only 22.2% had a GDS over 10 [15]. A GDS of 0-9 indicates “normal,” while a score of 10-19 correlates to “mild depression,” and 20-30 correlates to “severe depression.” An additional study found a significantly higher level of suicidal ideation, anxiety, and depression among patients with atopic dermatitis [15]. Incorporating TD as a means for geriatric patients to access dermatology presents a possible opportunity to reduce these barriers and may improve the QoL for the geriatric population.

Although there is ample evidence of TD’s effectiveness in a general outpatient setting, its application in the nursing home setting remains underexplored. A preliminary search of PROSPERO, the Cochrane Database of Systematic Reviews, and PubMed was completed and indicated no current systematic or scoping reviews on this topic. Published reviews related to the use of TD in nursing homes included comparisons between in-person dermatology and TD diagnoses [6] and the potential benefits of TD in triaging patients [12]. A related study, “Benefits of teledermatology for geriatric patients” [16], focused on the general geriatric population rather than nursing home residents. As such, this scoping review aims to (1) identify the most common dermatologic conditions diagnosed among nursing home residents and (2) identify the benefits tied to TD in addressing the needs of these diagnoses.

Overall, this scoping review will provide a comprehensive analysis of the benefits of using TD as a diagnostic and management tool for dermatologic conditions in the nursing home setting. Additional subtopics associated with the most prevalent TD diagnoses among nursing home residents will also be explored.

## Review

Materials and methods

Study Design

This scoping review was conducted to analyze the benefits of using TD for the diagnosis and management of dermatologic conditions, as well as a tool for improving the QoL of nursing home residents. Additional subtopics, including the most prevalent dermatologic diagnoses in nursing homes, were considered. A population, concept, and context framework was implemented to focus the research on (a) geriatric individuals receiving dermatologic care (population); (b) TD as a tool for diagnosis, management, and improved QoL (concept); and (c) nursing homes (context). Because TD research specific to elderly nursing home patients remains limited, this review includes TD benefit studies across all age groups and applies these findings to the nursing home context.

Protocol and Eligibility Criteria

A comprehensive search was conducted by three independent researchers using multiple databases, including Ovid MEDLINE, EMBASE, and Web of Science. To analyze the most common dermatologic diagnoses in nursing homes, the inclusion criteria included geriatric patients (>60 years old), nursing home patients, and studies published in English between 2015 and 2025. For analyzing the overall benefits of using TD, the inclusion criteria were identical except that dermatology patients of any age were eligible. Exclusion criteria for analyzing the most common dermatologic diagnoses in nursing homes and the benefits of using TD included articles that were older than 15 years and case reports.

Search Strategy

The search strategy was conducted using EMBASE, Ovid MEDLINE, and Web of Science, including articles from January 2015 to April 2025. Search terms combined controlled vocabulary (MeSH/Emtree) and keywords. Boolean operators (“AND”, “OR”) were also implemented. Table 2 outlines the results compiled by the database for top dermatology visits in nursing homes (2015-2025).

**Table 2:** Search strategy for identifying top dermatologic diagnoses in nursing home populations (2015-2025)

Database	Date searched	Query	Results
EMBASE	04/10/2025	#1 'dermatology':ab,ti,kw OR 'skin condition':ab,ti,kw OR 'skin diseases':ab,ti,kw (109,447) #2 'disease diagnosis'/exp OR 'medical diagnosis'/exp OR 'physical diagnosis'/exp (11,645,880) #3 #1 AND #2 (38,016) #4 'extended care facilities'/exp OR 'long-term care facilities'/exp OR 'nursing homes'/exp (87,008) #5 #3 AND #4	70
Ovid MEDLINE	04/10/2025	#1 (“dermatology” OR “skin condition” OR “skin diseases”) #2 ("disease diagnosis" OR "medical diagnosis" OR "physical diagnosis" OR "diagnoses") #3 #1 AND #2 #4 ("extended care facility" OR "long term care facility" OR "nursing home") #5 #3 AND #4	21
Web of Science	04/10/2025	#1 TS=(“dermatology” OR “skin condition” OR “skin diseases”) #2 TS=("disease diagnosis" OR "medical diagnosis" OR "diagnoses") #3 #1 AND #2 #4 TS=("extended care facility" OR "long term care facility" OR "nursing home”) #5 #3 AND #4	27

A second search was conducted to compile studies reporting the benefits of TD, which are outlined in Table 3.

**Table 3:** Search strategy for benefits of TD (2015-2025) TD, teledermatology

Database	Date searched	Query	Results
EMBASE	04/10/2025	#1 'teledermatology':ab,ti,kw OR 'tele-dermatolog*':ab,ti,kw OR 'virtual dermatology':ab,ti,kw OR 'store-and-forward dermatology':ab,ti,kw OR 'remote dermatologic care':ab,ti,kw (2,247) #2 'advantage'/exp (89) #3 'benefit' OR 'benefits' (1,504,675) #4 #2 OR #3 (1,504,762) #5 #1 AND #4	262
Ovid MEDLINE	04/10/2025	#1 ('teledermatology' OR 'tele-dermatolog*' OR 'virtual dermatology' OR 'store-and-forward dermatology' OR 'remote dermatologic care').ab,ti,kw. (1,554) #2 ('advantage' OR 'benefit' OR 'benefits').ab,ti,kw. (1,190,411) #3 #1 AND #2	156
Web of Science	04/10/2025	#1 TS=(“teledermatology” OR “tele-dermatolog*” OR “virtual dermatology” OR “store-and-forward dermatology” OR “remote dermatologic care”) (2,200) #2 TS=("advantage" OR "benefit" OR "benefits") (2,286,100) #3 #1 AND #2	221

Study/Source of Evidence Selection

All identified articles were uploaded into Rayyan (Rayyan Systems Inc., Cambridge, MA, USA), and duplicates were removed. Each independent reviewer screened all titles and abstracts, excluding those that did not align with our study’s inclusion criteria. After initial screening, full texts were uploaded to Google Drive (Google LLC, Mountain View, CA, USA) for further assessment. Reasons for exclusion were documented in the Preferred Reporting Items for Systematic reviews and Meta-Analyses (PRISMA) diagram, and any disagreements throughout the review process were resolved by the input of an additional reviewer. The selection processes for both searches were outlined in two separate PRISMA extension for scoping reviews (PRISMA-ScR) flow diagrams [17].

Quality Assessment

Quality assessment was performed using the Joanna Briggs Institute (JBI) Critical Appraisal Tools appropriate to each study design [18]. Articles were evaluated based on the proportion of applicable criteria met and categorized accordingly. All studies included in this review met the majority of JBI methodological quality criteria.

Results

Top Dermatology Diagnoses in Nursing Homes (PRISMA 1)

**Figure 1:**
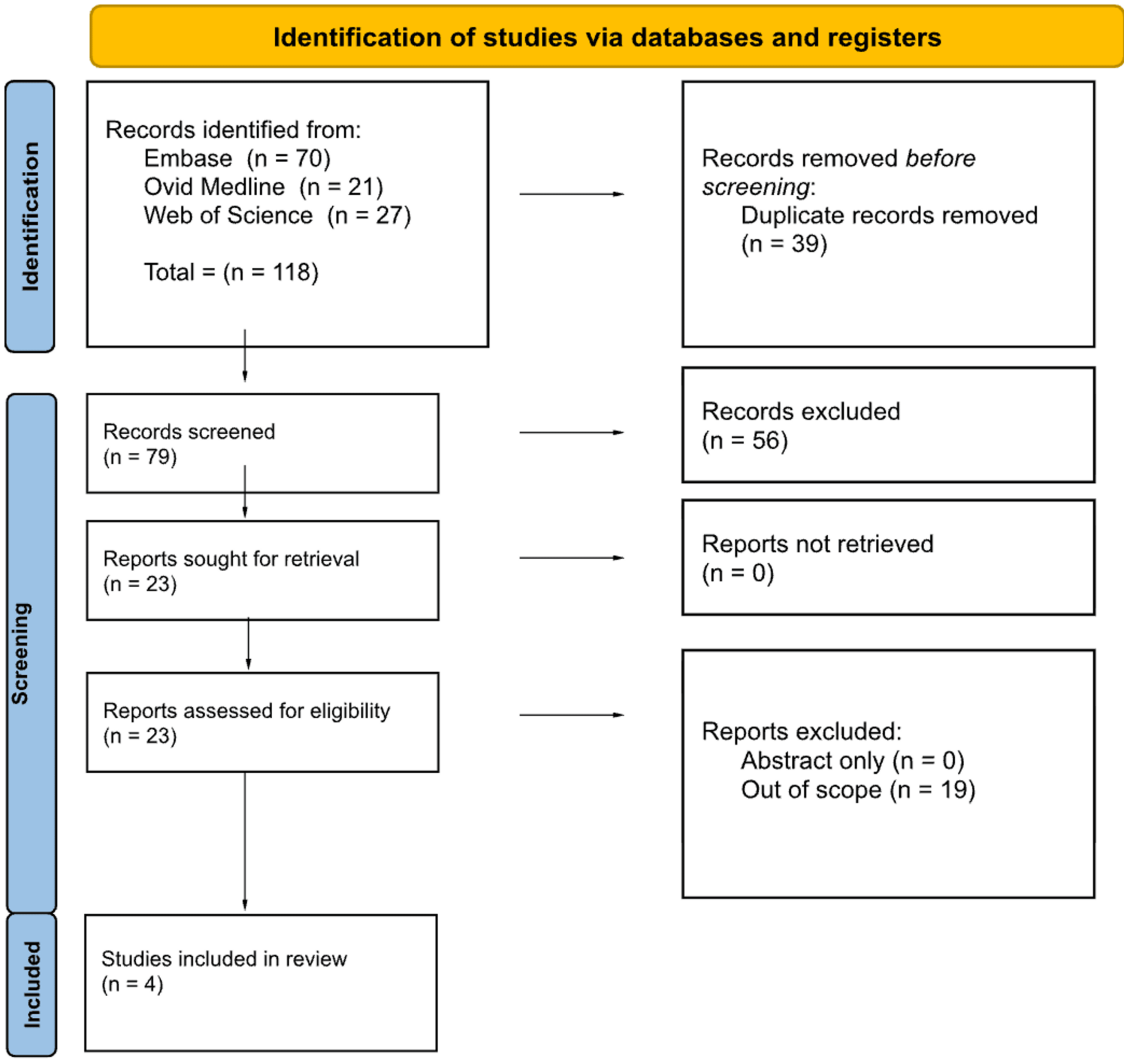
PRISMA diagram for the top dermatology diagnoses in nursing homes PRISMA, Preferred Reporting Items for Systematic reviews and Meta-Analyses

For top dermatology diagnoses in elderly nursing home patients, a total of 118 articles were identified. After screening, 23 articles were selected for full-text review, with four full-text articles meeting criteria for this review (Figure 1). Findings should be interpreted cautiously due to the limited study count and heterogeneity.

Moreover, the study characteristics for each article included in our analysis of top dermatology diagnoses in nursing homes are outlined in Table 4.

**Table 4:** Study characteristics of included articles for PRISMA 1 (n = 4) PRISMA, Preferred Reporting Items for Systematic reviews and Meta-Analyses

Author	Country	Study design	Population	Setting	Sample size
Klösters et al. (2022) [12]	Netherlands	Prospective multicenter observational study (02/18/15-02/11/21)	Residents of nursing homes receiving live dermatology consultations	Nursing homes	n = 270
Hahnel et al. (2017) [19]	Germany	Cross-sectional study	Residents of nursing homes in Berlin	Long-term care facilities	n = 223
Polat et al. (2017) [20]	Turkey	Cross-sectional study	Elderly residents	Two nursing homes	n = 105
Darjani et al. (2020) [21]	Iran	Cross-sectional study	Elderly residents (>60 years)	Charity nursing-home complex	n = 259

*Top Dermatologic Diagnoses in Nursing Home Patients*

Across the diagnosis-related studies, the five most frequent diagnoses included xerosis cutis (78-99%), tinea unguium (59-62%), seborrheic keratosis (31-56%), solar lentigines (35-90%), and eczema (17-37%) (Table 5).

**Table 5:** Summary of reported dermatologic diagnoses in nursing home populations

Author	Country	Benign tumor (%)	Premalignant (%)	Malignant (%)	Xerosis cutis (%)	Tinea unguium (%)	Seborrheic keratosis (%)	Eczema (%)	Pressure ulcers (%)	Solar lentigines (%)
Klösters et al. (2022) [12]	Netherlands	14.8	24.8	20	-	-	-	17.4	3	-
Hahnel et al. (2017) [19]	Berlin	-	-	-	99.1	62.3	56.5	-	-	-
Polat et al. (2017) [20]	Turkey	-	-	-	78.1	59	31.4	-	-	90.5
Darjani et al. (2020) [21]	Iran	68.3	-	-	-	-	15.4	37.5	-	35.9

**Figure 2:**
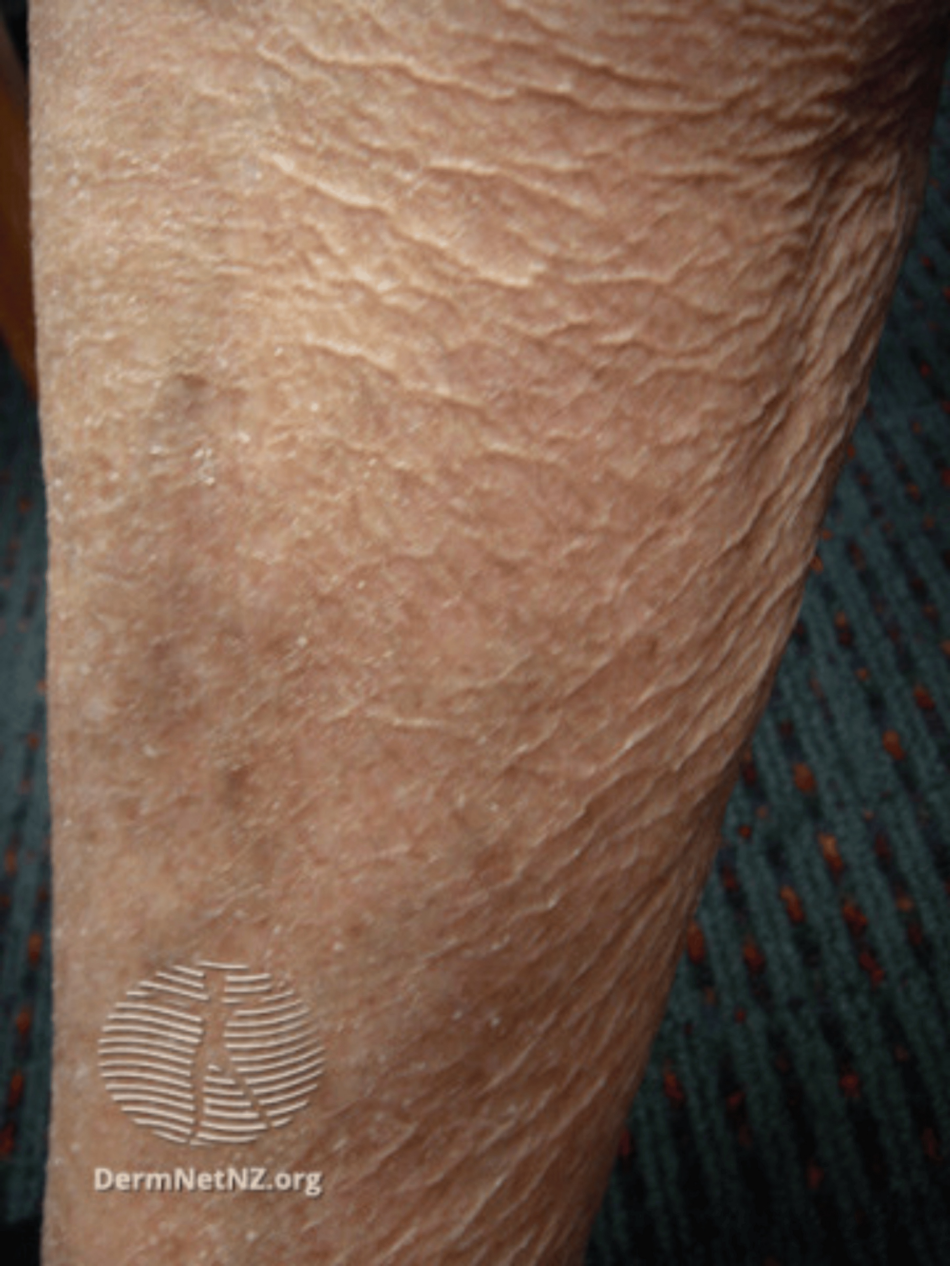
Xerosis cutis clinical presentation Image source: Gade et al. (2025) [22]; Creative Commons Attribution (CC BY) license

Xerosis cutis, with or without pruritus, was the most consistently reported condition across studies. The clinical presentation of this form of the diagnosis is portrayed in Figure 2.

**Figure 3:**
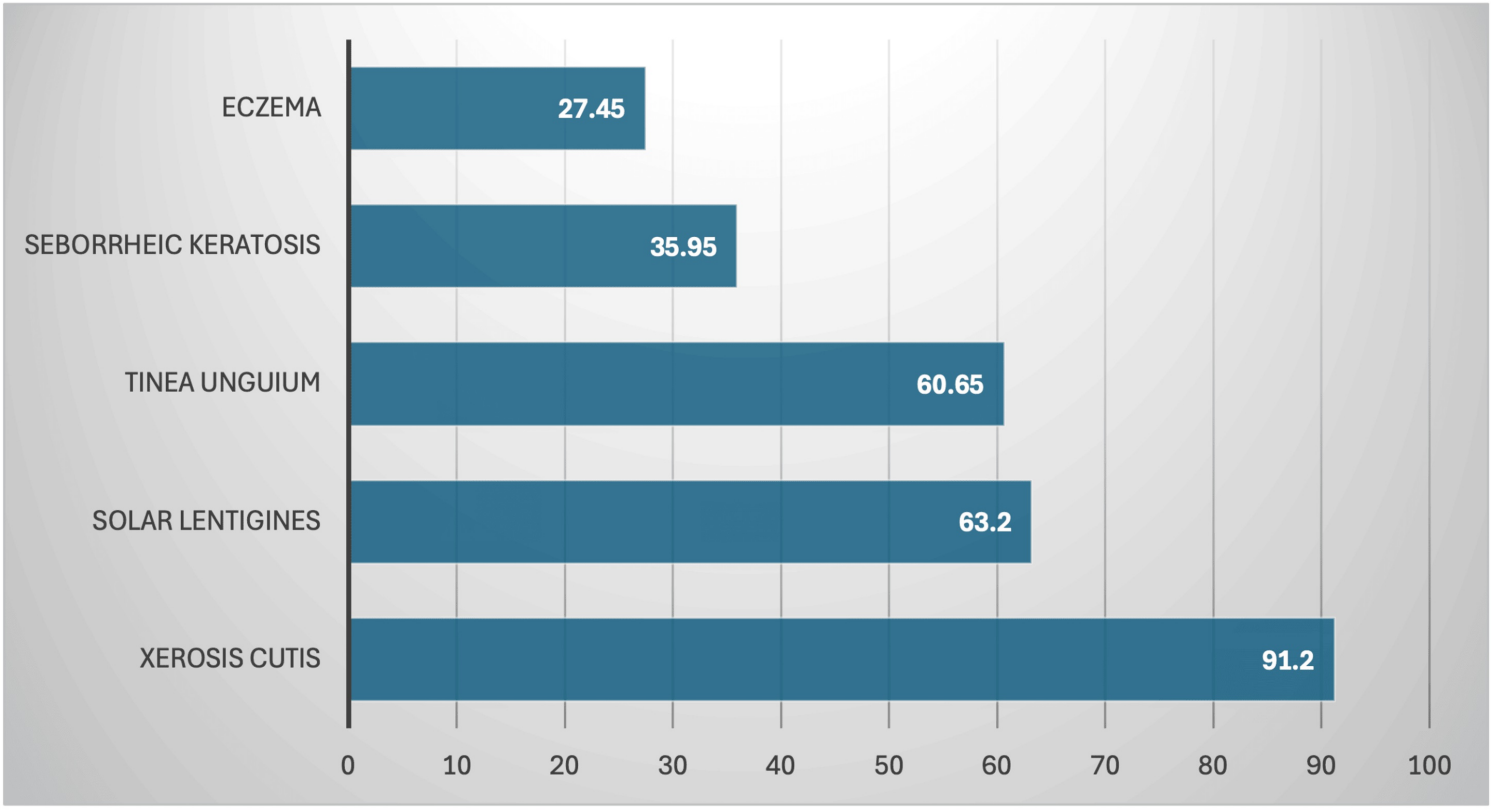
Mean prevalence of the most frequently reported dermatologic conditions among nursing home residents across included studies

The most frequent remaining diagnoses among nursing home patients are summarized in Figure 3 and include tinea unguium, seborrheic keratosis, solar lentigines, and eczema.

Benefits of TD (PRISMA 2)

**Figure 4:**
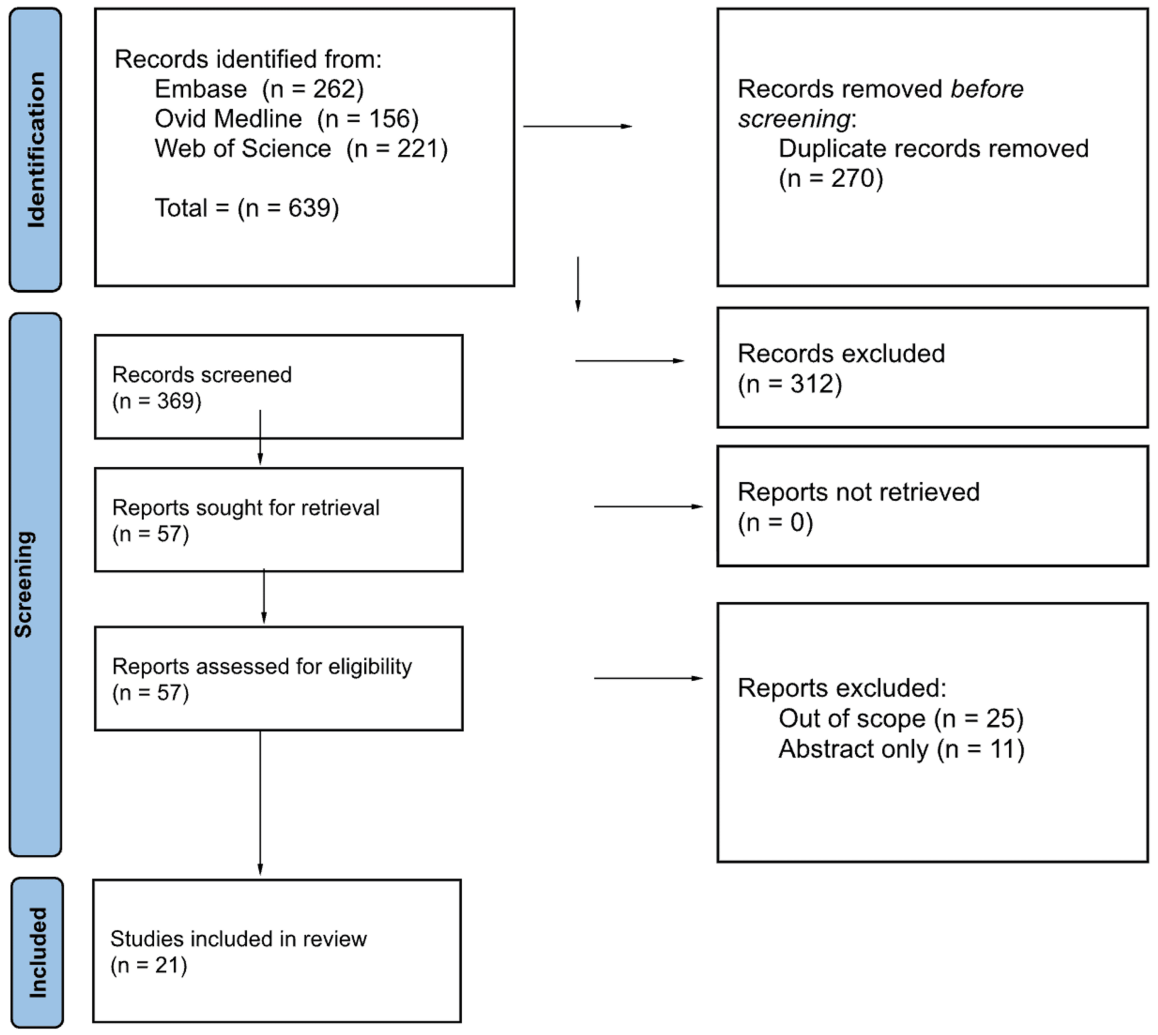
PRISMA diagram for the benefits of TD PRISMA, Preferred Reporting Items for Systematic reviews and Meta-Analyses; TD, teledermatology

Regarding the benefits of TD, a total of 639 articles were identified. After duplicate removal and screening, 57 articles were retrieved, and 21 met the inclusion criteria for this review (Figure 4).

Study Characteristics (for PRISMA 2)

The chosen studies regarding the benefits of TD included retrospective cohorts, systematic reviews, narrative reviews, economic evaluations, literature reviews, randomized controlled trials, and cross-sectional studies (Table 6).

**Table 6:** Study characteristics of included articles (n = 21) CA, cost analysis; CCH, collaborative connected health; CCT, controlled clinical trial; CEA, cost-effectiveness analysis; CMA, cost-minimization analysis; CUA, cost-utility analysis; NA, not applicable; RCT, randomized controlled trial; RT-TD, real-time teledermatology; SAF-TD, store-and-forward teledermatology; TD, teledermatology

Author	Country	Study design	Type of TD	Comparator	Population
Tommasino et al. (2024) [5]	Italy	Narrative review	SAF-TD	In-person care	Dermatology patients
Hindelang et al. (2024) [10]	Germany	Cross-sectional study	SAF-TD via the “OnlineDoctor” platform	None (observational)	Users ≥18 years submitting dermatologic surveys via the “OnlineDoctor” platform
Bianchi et al. (2020) [16]	Brazil	Retrospective cohort study	SAF-TD	In-person care	6,633 patients aged ≥60 years (12,770 lesions assessed)
Zakaria et al. (2019) [23]	USA	Retrospective pre- and post-cohort	SAF-TD (Medweb)	Pre vs. post-TD	Dermatology patients
Wallace et al. (2024) [24]	USA	Retrospective cohort	SAF-TD	In-person care	Dermatology patients
Van Enst et al. (2024) [25]	Netherlands	Systematic review (five RCTs and one CCT)	Mixed modalities - SAF-TD video consultation	In-person care	Chronic inflammatory skin conditions
Tognetti et al. (2021) [26]	Italy	Narrative review	Mixed modalities - SAF-TD, hybrid, and tertiary teleconsultations	NA	Dermatology patients
Snoswell et al. (2016) [27]	Australia	Systematic Review (one CA, four CMA, four CEA, and two CUA)	SAF-TD	In-person care	Dermatology patients
Skinner et al. (2022) [28]	USA	Economic evaluation (Markov model)	CCH model	In-person care	Psoriasis patients
Coates et al. (2015) [29]	USA	Narrative review	Mixed modalities - SAF-TD, RT-TD, and hybrid	In-person care	Dermatology patients
Pala et al. (2020) [30]	Poland	Systematic literature review: >30 studies (2000-2018)	SAF-TD	In-person care	Cutaneous melanoma patients
Mehta et al. (2025) [31]	Canada	Systematic review (21 studies, 2009-2023)	Mixed modalities - asynchronous (mobile photos/web portals) and synchronous (video visits)	In-person care	Patients with acne vulgaris
Martora et al. (2023) [32]	Italy	Systematic review (92 studies)	Mixed modalities - synchronous (video calls) and asynchronous (images, messaging, and phone calls)	In-person care	Chronic inflammatory skin conditions
Lopez-Villegas (2025) [33]	Spain	Randomized, controlled, non-blinded multicenter clinical trial	SAF-TD	In-person primary care	Dermatology patients > 18 years
Lee et al. (2018) [34]	Australia	Literature review (2015-2017)	Mixed modalities - SAF-TD, RT-TD, and mobile teledermoscopy	In-person care	Dermatology patients
Hadeler et al. (2021) [35]	USA	Narrative review	Mixed modalities - synchronous (video visits) and hybrid (photo + live video) TD	In-person care	Underserved communities
Sud and Anjankar (2022) [36]	India	Narrative review	SAF-TD	In-person care	Dermatology patients
Bodle et al. (2022) [37]	Switzerland	Systematic review (13 included studies)	SAF-TD	In-person care	Patients with acne vulgaris
Benedit and Aycock (2022) [38]	USA	Narrative review	Mixed modalities - SAF-TD, RT-TD, and mobile app-based TD	In-person care	Rural residents with melanoma
Ahuja et al. (2022) [39]	USA	Narrative review	Mixed modalities - SAF-TD, RT-TD, and hybrid TD models	In-person care	Rural, urban, underserved, and globally isolated populations
Almaziad et al. (2021) [40]	Saudi Arabia	Cross-sectional survey	TD	In-person care	Practicing Saudi dermatologists

Across the TD benefit studies, cost-effectiveness, accessibility, diagnostic accuracy and concordance, patient satisfaction, and clinical outcomes were found to be consistently reported. These findings are summarized in Table 7, Table 8, Table 9, Table 10, and Table 11.

**Table 7:** Cost-effectiveness outcomes of TD FTF, face-to-face; TD, teledermatology

Study	Metric	Key cost-related findings
Wallace et al. (2024) [24]	Travel cost savings	TD spares travel burden and expenses.
Tognetti et al. (2021) [26]	Community cost savings; system-level savings	Countries have recently implemented full or partial reimbursements to those using TD.
Snoswell et al. (2016) [27]	Travel cost savings; patient cost savings	TD increases cost-effectiveness in populations requiring further travel for FTF care.
Skinner et al. (2022) [28]	System-level savings	TD for psoriasis could lower US medical costs by an estimated $1.5 billion over five years.
Lopez-Villegas et al. (2025) [33]	Patient cost savings	Patients in the TD group reported cost savings of 77.59%.
Lee et al. (2018) [34]	Travel cost savings; avoided in-person visits	Patients using TD benefited economically due to decreased FTF specialist referrals and decreased travel time.
Benedit and Aycock (2022) [38]	Travel cost savings	Patients using TD avoid expenses related to travel.
Ahuja et al. (2022) [39]	Patient cost savings	TD brings increased savings to areas with limited dermatological care.

**Table 8:** Access to care outcomes of TD FTF, face-to-face; SAF-TD, store-and-forward teledermatology; TD, teledermatology

Study	Metric	Key access-related findings
Bianchi et al. (2020) [16]	Wait times	Mean wait time for FTF decreased by 78% using TD as a triaging tool.
Zakaria et al. (2019) [23]	Wait times	New patient wait times significantly decreased from 84.6 to 6.7 days (p < 0.001).
Wallace et al. (2024) [24]	Time to follow up	Time to follow up decreased from 73.5 to 58 days.
Coates et al. (2015) [29]	Wait times	Wait times decreased in rural areas with limited ability to meet with geographically distant providers.
Lee et al. (2018) [34]	Time to appointment	Use of TD as a triaging tool reduced FTF referrals by 31%, improving time to appointment.
Sud and Anjankar 2022 [36]	Time to appointment	SAF-TD results in an average reduction of 45.5-61.5% in FTF visits, facilitating faster access to care.
Benedit and Aycock 2022 [38]	Wait times	TD reduces wait times for rural skin cancer patients.
Ahuja et al. (2022) [39]	Wait times	Enhanced triaging decreases wait times.
Almaziad et al. (2021) [40]	Time to appointment	Six out of 11 studies found TD to be beneficial as a triaging tool in both inpatient and outpatient settings.

**Table 9:** Diagnostic accuracy and concordance outcomes of TD FTF, face-to-face; SAF-TD, store-and-forward teledermatology; TD, teledermatology

Study	Metric	Key diagnostic accuracy findings
Tognetti et al. (2021) [26]	Diagnostic and management concordance	Average concordance rate of 91% in a group of 391 patients with a total of 30 skin conditions.
Coates et al. (2015) [29]	Diagnostic and management concordance	SAF-TD has a high degree of complete concordance and an even higher degree for partial concordance.
Pala et al. (2020) [30]	Diagnostic and management concordance	TD had high concordance rates for diagnosis and management.
Lee et al. (2018) [34]	Diagnostic and management concordance	Concordance rates ranged from 45% to 96% for diagnosis and from 66% to 96% for management.
Sud and Anjankar (2022) [36]	Diagnostic and management concordance	TD data have supported the accuracy of diagnosis and management.
Ahuja et al. (2022) [39]	Diagnostic and management concordance	FTF and TD had high diagnostic concordance.
Almaziad et al. (2021) [40]	Diagnostic and management concordance	High concordance between FTF and TD diagnosis and management.

**Table 10:** Patient satisfaction outcomes of TD TD, teledermatology

Study	Metric	Key patient satisfaction findings
Hindelang et al. (2024) [10]	Patient preference	Among 1,141 patients, 77.6% indicated a preference for TD for future dermatologic visits.
Coates et al. (2015) [29]	Patient satisfaction	Satisfaction rates were high, especially in rural populations.
Lee et al. (2018) [34]	Patient attitudes	Attitude to TD reported to be good due to comfort and privacy.
Sud and Anjankar (2022) [36]	Patient satisfaction	Satisfaction rates correlate with reduced wait times and increased communication with providers.
Bodle et al. (2022) [37]	Patient satisfaction	73.9-75.6% of patients reported satisfaction with TD.
Benedit and Aycock (2022) [38]	Perceived convenience and privacy	Patient surveys reported better privacy and improved comfort.
Ahuja et al. (2022) [39]	Patient satisfaction	Underserved patients reported improved satisfaction due to reduced access barriers.
Almaziad et al. (2021) [40]	Perceived safety	Majority satisfied; 69% reported that TD reduced infection risk.

**Table 11:** Clinical outcomes of TD FTF, face-to-face; DLQI, Dermatology Life Quality Index; PASI, Psoriasis Area and Severity Index; POEM, Patient-Oriented Eczema Measure; QoL, quality of life; RCT, randomized controlled trial; TD, teledermatology

Study	Metric	Key clinical outcome findings
Zakaria et al. (2019) [23]	Clinical efficiency	Efficiency increased from 2.27 to 2.63 cases/h (p = 0.010).
Van Enst et al. (2024) [25]	Disease activity (PASI); QoL (DLQI and POEM)	RCT data revealed equivalence in PASI and DLQI using TD
Benedit and Aycock (2022) [38]	No-show rates	TD reduces no-shows compared to FTF from 84% to 24%.
Ahuja et al. (2022) [39]	No-show rates	Minorities using TD had a lower no-show rate.

Cost-Effectiveness

Across the TD benefit studies, eight studies highlighted a general trend toward reduced costs associated with TD, including factors such as patient savings, community savings, and travel savings. Specifically, TD was tied to a decrease in community care costs, a reduction of $1,076,000, and patient travel costs, with a reduction of $136,531 [25]. In particular, one article quantified a four-hour decrease in travel time per patient [25]. This number increases among patient populations with limited mobility or those residing in rural areas, who may have to travel farther for in-person access to care.

Access to Care

Accessibility was found to increase across nine of the 21 TD benefit studies, as measured by wait time to appointment and time to follow-up. One study found a decrease in the average new patient wait time using TD from 84.6 to 6.7 days (p < 0.001), likely due to its implementation in patient triage and quick turnaround time [23].

Diagnostic Accuracy

Seven out of the 21 TD benefit studies highlight a high concordance rate between TD diagnoses and in-person consultations, with two studies reporting rates of 91-97% [26,30]. The high accuracy of TD as a diagnostic platform allowed for a reduction of in-person referrals by 31-88% [34]. Almaziad et al. reported a high concordance rate between FTF and TD not only in diagnosis but also in the management of skin conditions [40].

Patient Satisfaction

Patient satisfaction outcomes were mainly positive across eight studies out of the 21 TD benefit studies included. Patients reported reduced wait times, better convenience, and higher satisfaction with TD compared to in-person visits. One study quantified patient satisfaction as 89.1% satisfied overall and 95.1% satisfied due to convenience [10]. Two studies also highlighted perceived patient safety, with decreased infection risk and no significant increase in adverse effects [34,40]. Satisfaction significantly increased among patient populations with reduced access to care, such as rural and nursing home populations [29,34,39].

Clinical Outcomes

Clinical outcomes assessed in the TD benefit studies included disease activity (Psoriasis Area and Severity Index (PASI) and Scoring Atopic Dermatitis), QoL (Dermatology Life Quality Index (DLQI) and Patient-Oriented Eczema Measure), effective disease monitoring, treatment adherence, and reduced no-show rates. Three studies out of the seven that discussed clinical outcomes consistently found that reduced no-show rates and efficiency were improved with TD as compared with FTF, ranging from 84% to 24% [23,38,39]. One study found an equivalence in PASI and DLQI scores between the two modalities [25].

Discussion

The purpose of this scoping review was twofold: to identify the most common dermatologic diagnoses in the nursing home population and to evaluate the benefits of TD in addressing these concerns. Our findings revealed that TD enhanced diagnostic accuracy, accessibility, and continued care among elderly patients. Rapid diagnoses allow for faster relief, decreased in-person referrals, and improved overall clinical outcomes. TD has been shown to have high diagnostic concordance with FTF consultations, which is especially advantageous for patients with limited mobility. In nursing home settings, TD offers a cost-effective approach to managing dermatological conditions without the added burden of travel and allows for in-person follow-up only when needed. In terms of access to care, more efficient triage may reduce wait times for patients. This overall efficiency reduces the time to an appointment, which may be a feature of traditional FTF dermatology. Patients are often satisfied with TD due to increased privacy and communication, fewer workdays lost, and improved access to care. This becomes apparent in the decreased no-show rate for TD appointments. In addition, clinical outcomes, including disease activity, have been reported to be equivalent between the two modalities. These findings align with current literature highlighting the growing utility of TD among geriatric patients while emphasizing the specific benefits for nursing home patients.

Xerosis cutis, or abnormally dry skin, is one of the most common skin conditions seen within the elderly population. The skin’s ability to retain moisture decreases with age, which is considered a multifactorial process. Changes in intercellular lipid levels, water metabolism, sebum production, and keratinization contribute to transepidermal water loss and thus to weakening of the skin barrier [41]. Clinically, xerosis cutis can present with pruritus, scaling, and discomfort, typically on the extensor surfaces of the lower extremities. Women are more commonly impacted by the condition as compared with their male counterparts, as men have higher and more stable sebum production over the course of their lives [41].

Xerosis cutis represents a significant geriatric public health concern, especially among nursing home patients, where it is highly prevalent [42]. This condition is often overlooked in nursing homes for many reasons. Skin issues may be neglected, as they are not the primary reason nursing home patients seek care. In addition, caregivers within nursing facilities are not professionally trained to perform skin inspections to identify these conditions [42]. This gap emphasizes the need for TD and its promising impact.

Dermatologic conditions such as xerosis cutis have substantial impacts on patients’ QoL. The comorbidities associated with many of these skin conditions include mental health disorders such as anxiety and depression. In a 2022 study on the mental health burden of 127 patients diagnosed with xerosis cutis, there was a significant correlation found between the condition and a decreased QoL (p = 0.041), higher anxiety (p = 0.029), and increased dysmorphic concerns due to their appearance (p < 0.001) [14]. The high prevalence of pruritus associated with xerosis cutis increases the risk of secondary skin infections due to scratching, which creates an entry path for additional pathogens [42]. Additionally, associated pruritus may interfere with patients’ sleep and daily activities. Many variables associated with aging, such as decreased collagen synthesis and sebaceous secretion, as well as a weakened epidermal barrier, increase the susceptibility of the geriatric population to these skin disorders [42].

Future Directions

TD has continued to evolve within the clinical care setting, as it has shown rapid growth and uptake, especially since the COVID-19 pandemic. The implementation of AI in conjunction with TD may also prove to be promising, mirroring broader advancements in AI across various fields. Future studies focusing on AI within TD, particularly regarding its impact on the geriatric community, could significantly expand its scope and efficacy. This could lead to more efficient and effective diagnosis and treatment of dermatologic conditions in nursing homes, benefiting a vulnerable population. 

Limitations

Despite its strengths, this scoping review is subject to limitations. While the inclusion criteria for identifying the top diagnoses in nursing home residents limited subjects to those aged 60 years and older, the same age criteria were not met when evaluating the top benefits of TD. Due to the narrow scope of existing literature on TD specifically within elderly nursing home populations, we broadened our approach to focus on studies reporting the most common benefits of using TD across any age group and applied these findings to the nursing home population. Future research may choose to delve more into the benefits specifically within this demographic.

## Conclusions

Attending in-person dermatology visits can be difficult for elderly nursing home patients due to physical or cognitive barriers. Telehealth, and TD in particular, may represent an important advancement in the care of these patients, as it has been associated with earlier diagnosis and management of skin conditions that may otherwise become mental, physical, or financial burdens. Beyond diagnosis, TD may also support ongoing care for patients with chronic dermatologic issues. Overall, findings from this scoping review suggest that TD is a promising and efficient approach for the future of healthcare, especially for elderly patients within nursing homes. However, the existing evidence specific to nursing home settings remains limited. Continued improvements in imaging and communication technologies may further enhance diagnostic accuracy and potentially reduce the need for additional in-person evaluations. As telehealth regulations evolve, it is essential to highlight the wide range of benefits TD provides. Recognizing these advantages will help efforts to sustain and expand its use, ultimately improving access to high-quality dermatologic care for nursing home residents and other vulnerable populations.
